# Supplementary material for: Are carbon emissions trading and green financial instruments synergistic? -Comprehensive quantitative research based on content analysis
Source: PLoS One. 2024 Mar 7;19(3):e0298601. doi: 10.1371/journal.pone.0298601 (PMC10919601; doi:10.1371/journal.pone.0298601)
Supplement: S1 Data — (ZIP) [file pone.0298601.s001.zip › Data/地级市Z_F工作报告.zip!/░▓╗╒/╦▐╓▌╩╨/└┤╫╘╣1⁄2╓┌║┼í╛┬φ┐╦╩2╛▌═°í┐_2006.docx]

2006年2月8日宿州市第二届人民代表大会第三次会议

市 长  唐承沛

各位代表：

现在，我代表宿州市人民政府，向大会作政府工作报告，请予审议，并请市政协委员和其他列席人员提出意见。

一、2005年及“十五”工作回顾

过去的一年，在中共宿州市委的领导下，全市人民紧紧围绕“抢抓机遇、乘势而上、奋力崛起”的总体要求，牢牢把握第一要务，积极适应宏观调控，努力破解发展难题，较好地完成了市二届人大二次会议确定的各项任务。

整体经济保持较快增长。全市生产总值完成313亿元，增长8.3%。农村经济受到局部灾害影响略有下降，粮棉油因灾减产，瓜果蔬菜和畜牧产品增效显著；农业产业化水平不断提高，省级产业化龙头企业达到10家；劳务输出102万人，比上年净增14.8万人。工业经济快速增长成为最大亮点，规模以上工业增加值增长30.1%，工业用电量增长18%，均居全省前列；骨干企业支撑作用明显，年销售收入超亿元的企业达到17家，其中超5亿元的企业达到5家。财政收入完成13.4亿元，按可比口径增长19.3%。金融机构存贷规模双增，不良贷款比率下降10个百分点。社会消费品零售总额达到85.4亿元，增长11.6%，创近年来最高纪录。

项目建设取得重大突破。深入实施项目带动战略，全面启动“6＋3”工程，全社会固定资产投资完成85亿元，增长25.5%。在建和新开工千万元以上项目233个，比上年增加67个。汇源电厂#4机组、砀山果蔬罐头、鲁皖成品油管道宿州段等一批项目建成；华电宿州电厂一期、锦丰食品二期等项目建设加快；交通基础设施完成投资5.2亿元，建设里程608公里。华电宿州电厂二期、丰原生物化工项目、泗永和睢明高速公路、宿淮铁路等重大项目前期工作扎实推进。

东向发展开局良好。坚持把融入徐州经济圈作为东向发展的突破口，把实施“跟跑”策略作为提升县区发展水平的重要举措，与徐州市政府签署了全面合作框架协议，两市合作取得良好开端。坚持不懈地开展小分队招商、驻点招商，成功举办了温州、台州项目推介签约会和上海对台商投资环境说明会，招商引资规模持续扩大。全年实际到位内资45亿元，增长47.5%。实际利用外资2720万美元，增长16.7%。进出口总额完成4945万美元，增长90%。宿州经济开发区建设快速推进，新开工项目30个，实际到位资金6亿元。泗县、灵璧、萧县开发区在规范中加快发展，埇桥、砀山经济开发区开始启动。

城市面貌变化显著。城市规划得到加强，宿城总体规划调整、沱河景观规划、L型新区控制性详规相继完成。城市基础设施建设步伐加快，外环路主路面全线贯通，银河一路、银河二路、张仙庙路南段、汴河西路、宿怀路和东昌路改造全面竣工，雪枫广场、运粮河景区正式开放。政务新区建设初具雏形，行政中心即将交付使用。城区小街小巷五年整治计划进展顺利，改造街巷17条。城市综合开发势头强劲，在建房地产面积277万平方米，竣工85.9万平方米。创建省级文明城市工作先进市取得阶段性成果，城市管理水平进一步提升。

县域经济竞相发展。各县区坚持加快发展不动摇，正视困难，不甘落后，奋发有为，强力推进招商引资和项目建设，县域经济呈现出争先进位、蓬勃发展的良好局面。大开放的氛围日益浓厚，经济活力明显增强，部分主要经济指标保持了较高增速，招商引资均超额完成目标任务，砀山、萧县、泗县规模以上工业增加值增速超过全市平均水平。特色经济亮点突出，灵璧、砀山分别被省政府确定为农业标准化和农业产业化示范县，埇桥区跻身全省蔬菜十强县行列，萧县荣获“中国防腐蚀业第一县”称号。

和谐宿州建设扎实推进。群众生活进一步改善，城镇居民人均可支配收入7910元，增长12.8%；农民人均纯收入2156元，与上年持平略有增长。就业和社会保障工作力度加大，城镇登记失业率控制在3.9%以内，“两个确保”继续巩固，低保人均月补差提高12元。科技创业服务中心基本建成，科技创新及成果转化工作不断加强。全市高考本科上线人数稳居全省前列，萧县一中、泗县一中升格为省级示范高中。疾病预防控制体系和医疗救治体系建设进展顺利，高致病性禽流感得到有效防控。一批文艺创作和剧目在国内赛事中获奖，泗州戏及萧县民间书画、灵璧钟馗画分别被确定为国家和省级非物质文化遗产。计划生育、国土资源、环境保护等基本国策得到较好落实。国防、人防建设卓有成效，双拥优抚工作在全省领先。人事制度改革稳步推进。第一次经济普查取得阶段性成果。广播电视、新闻出版、民族宗教、外事侨务、审计、物价、体育、气象、地震、对台、档案、红十字会、地方志等工作迈出新步伐。强化社会治安综合治理，认真负责地处置公共突发事件，依法有序畅通群众诉求渠道，社会大局总体稳定。切实抓好生产救灾和扶贫开发，灾区群众和社会弱势群体得到妥善救助。妇女、儿童、老龄和残疾人事业取得新进展。

民主法制和政府自身建设全面加强。“四五”普法工作顺利通过验收。基层民主建设深入推进，第六届村民委员会换届选举圆满完成。深入贯彻《行政许可法》，成立了市政府法律顾问组，取消行政许可项目116项，行政审批和行政性收费进一步规范。自觉接受人大、政协和群众监督，高质量办结市人大代表议案2件、建议72件，市政协委员提案166件。广泛听取民主党派、工商联、无党派人士的意见，与工会、共青团、妇联等人民团体的联系进一步密切。反腐倡廉深入开展，政风建设取得积极成效。

各位代表，2005年发展任务的完成，标志着全市“十五”计划的全面实现。五年来，我们坚持以解放思想为先导，始终保持发展战略的连续性、稳定性和开拓性，强力推进加快发展、富民强市的各项工作，先后战胜非典疫情和特大洪涝灾害，积极应对宏观调控影响，促进了经济社会快速健康发展。全市生产总值连跨两个百亿元台阶，年均增长8.6%；全社会固定资产投资年均增长15%，招商引资规模翻了三番，一批事关发展全局的重大项目相继开工；城市路网全面启动，服务功能日趋完善，城市面貌日新月异；体制机制创新向纵深推进，人民生活水平进一步提高，全市经济实力、发展环境、支撑条件发生了根本性变化。五年来，我们在不断深化市情认识的基础上，规划了“两个跃升、一个崛起”的奋斗目标，确立了以大开放为主战略，以结构调整为主线，以工业化、城市化为核心的发展思路，作出了实施“6＋3”工程、融入徐州经济圈、集全市之力建设经济开发区等一系列重大决策，凝聚了广大干部群众的共识，全市上下求富思变、奋力崛起的氛围已经形成，为未来发展奠定了坚实基础。五年来，全市广大干部群众艰苦创业，奋勇开拓，宿州所取得的每一个进步，都包含着全市干部群众的辛劳与智慧，是全市人民与时俱进、创新创业丰硕成果的具体体现。在此，我谨代表市人民政府，向全市广大工人、农民、知识分子和离退休老同志，向各民主党派、工商联和人民团体，向驻宿解放军、武警官兵和政法干警致以崇高的敬意！向所有参与、支持、帮助宿州发展的海内外朋友，表示衷心的感谢！

各位代表，过去五年的发展成就令人振奋，对存在问题也丝毫不能忽视。经济结构性矛盾突出，第一产业比重过高，经济发展规避市场风险、抵御自然灾害的能力不强；开放型经济、非公有制经济发展水平偏低，影响发展的体制性障碍亟待解决；基础设施和公共服务薄弱，发展滞后所积累的各种社会矛盾日益凸显，构建和谐社会的任务相当艰巨；少数职能部门管理越位、监管缺位的问题交叉并存，部分干部责任意识淡薄、事业心不强、作风不实等现象还比较突出。我们必须时刻保持清醒的头脑，切实增强责任意识和忧患意识，以对人民群众高度负责的精神，努力改进工作，不断实现新的发展。

二、“十一五”发展的形势与任务

“十一五”时期，是我市乘势而上、奋力崛起、全面建设小康社会的重要阶段，是我们必须紧紧抓住，并且可以大有作为的战略机遇期。未来五年，宏观经济继续保持较快发展，国际国内产业调整加速，有利于我市大规模承接产业转移，加快对外开放步伐；国家积极促进中部崛起，建设社会主义新农村，省委、省政府实施东向发展战略，重点支持毗邻苏浙地区率先突破，我们面临着难得的发展机遇。未来五年，是我市工业化、城市化快速提升的关键时期。全市人均生产总值将突破1000美元，第二产业逐步赶上并超过第一产业比重，三次产业结构将实现重要突破，经济发展形态将由以农业为主向工业主导型转变，城市建设将由低水平的半城半乡向区域性现代化中心城市转变，整体发展面临着从艰难爬坡向奋力崛起的重要转折。未来五年，是我市多年积累的能量加速释放，全民创业的活力竞相迸发，加快发展的氛围更加浓厚的五年，也是全市综合经济实力显著提升，努力实现城乡统筹发展、经济社会统筹发展的五年，发展的成果将更多地惠及百姓，人民群众的生活水平、生活质量将大幅度提高，和谐宿州建设将迈出新的步伐。尽管在前进的道路上，我们还将受到外部不确定因素的影响，面临着日趋激烈的区域竞争压力，但我们只要充分利用好战略机遇期，宿州就一定能够在新的起点上实现更快更好的发展。

“十一五”经济社会发展的指导思想是：认真落实科学发展观，坚持发展是硬道理，坚持以农业为基础，以大开放为主战略，以结构调整为主线，以工业化、城市化为核心，进一步突出东向发展、工业强市、项目支撑、中心城市带动和区域协调发展

等战略重点，扩张经济总量，提升经济质量，统筹城乡发展，加快建设和谐宿州，全面实现“两个跃升、一个崛起”的奋斗目标。

“十一五”发展的主要任务是：

——保持经济快速平稳增长，力争经济总量翻一番

坚持把发展作为解决一切问题的关键，进一步强化速度意识，加快发展步伐，力争到2010年，全市生产总值当年价超过600亿元，比2005年翻一番，年均增长11%，人均生产总值达到1200美元；财政收入保持同步增长；全社会固定资产投资年均增长25%，一批牵动性项目相继建成；经济结构实现重要突破，三次产业比例力争调整到30:40:30；工业增加值年均增长25%左右，其中规模以上工业增加值年均增长30%左右，实现三年倍增。在保持经济快速发展的同时，更加注重经济增长方式的转变，推进科技进步，提升自主创新能力，增强经济运行的稳定性和协调性，走科学发展、和谐发展之路。

——深入推进东向发展，全面提升对外开放水平

坚持更加主动的大开放战略，以融入徐州经济圈为起跳板，积极开展全方位、多层次的对外交流与合作，努力形成与长三角地区互动发展的良好局面。鼓励支持各县区以苏北毗邻县为标杆，在发展环境、服务效率、优惠政策等方面开展竞争，力争用三年左右时间，使经济发展速度达到苏北平均水平，在省内位次明显前移。强势推进招商引资，坚持扩大规模与提高质量相结合，承接产业转移与推动产业升级相结合，引资与引智相结合，围绕资源深度开发、国有企业改组改造、基础设施和生态建设，积极引进战略投资伙伴和基地型、龙头型项目，确保五年实际利用内资突破300亿元，利用外资突破1亿美元。坚定不移地建设各类开发区和工业园区，加快设施配套，创优服务环境，创新体制机制，加快要素集聚，提升开发区产业层次和运作水平，形成一批特色鲜明的产业集群和块状经济。

——实施工业强市战略，促进产业结构优化升级

坚持把实施工业突破作为奋力崛起的根本途径，着力培育工业发展的内生机制，不断改善工业发展的外部环境，切实增强工业对经济发展的支撑力。立足现有资源和产业基础，着力打造煤电能源、加工制造、农副产品深加工三个百亿元产业基地，构建优势产业体系。坚持扶优扶强，推进企业战略性重组，培植一批主业突出、核心竞争力强的企业集团，力争到2010年，年销售收入超过5亿元的企业达20家以上，其中5～10亿元的企业10家，超10亿元的企业10家，超20亿元的企业3家。高度重视中小企业发展，建立健全有利于中小企业发展的制度和服务平台，形成一批“专精特新”的中小企业集群，力争到“十一五”末，全市规模以上工业企业超过400家。

——坚持城乡互动，推动社会主义新农村建设

坚持以增加农民收入为核心，紧紧围绕“生产发展、生活宽裕、乡风文明、村容整洁、管理民主”的要求，把建设社会主义新农村的各项任务落到实处。加快推进农业产业化，建设一批国家级和省级龙头企业。发展各类农民合作经济组织，加强农村基层供销社改造，提高农民组织化程度。加快农业结构调整，提高畜牧、水果、蔬菜、林业基地建设水平。努力打造宿州劳务品牌，大力发展劳务产业。加强镇村规划和耕地保护，加快“空心村”整治，促进农村面貌整体改观。全面推进农村综合改革，完成乡镇机构、县乡财政体制、农村义务教育改革任务，建立农村工作新机制。强化政府对农村的公共服务，围绕“水、电、路、医、学”，加大对农业和农村基础设施的投入，加强扶贫开发工作，力争到“十一五”末，村村实现油路相通，人畜饮水安全得到基本保障，农村义务教育“两免一补”政策全面落实，新型农村合作医疗制度基本建立，健康、文明、科学的生活方式蔚然成风，农民的文明素质明显提高。

——突出项目支撑，打造奋力崛起的坚实平台

坚持把项目建设作为加快发展的重要抓手，以项目为载体，培育新的经济增长点，增强发展后劲。紧紧围绕“6＋3”工程，按照经济社会发展要求，加强与国家产业政策、发展规划的对接，策划一批事关全局的重大工程、重大项目，始终保持“6＋3”工程1000亿元的项目储备规模。认真研究大企业的投资动态，依托资源优势和产业基础，加快实施一批重大工业项目，进一步提升产业层次，培育主导产业。优化重大基础设施布局，加快铁路、高速公路建设和国道、省道、航道升级改造，完成“两纵两横”高速公路网建设，构建高速便捷的现代综合交通网络。加强水利设施配套，争取实施淮水北调、新汴河调水工程，增强水资源保障能力。完善项目工作机制，夯实工作基础，强化项目落地，始终保持投资强劲增势，确保从2006年开始，每年全社会固定资产投资超过100亿元，五年累计达到870亿元。

——强化中心城市带动，努力培育区域增长极

坚持以提高城市承载力和产业支撑力为核心，人口扩张与产业发展相适应，基础设施建设与城市品位提升相促进，充分发挥中心城市对区域经济发展的辐射带动作用。按照“一疏散、两集中”的要求，完善L型新区，建设发展好老城区，启动建设汴北新城区，实现跨新汴河发展。加大城市基础设施投入，集中力量建设一批骨干工程，全面完善城市路网，提升综合服务功能，塑造城市特色，打造适宜人居的城市环境。力争到“十一五”末，宿城建成区规模达60KM2，城市人口达60万左右。加强县城规划与建设，加快扩张县城规模，力争各县城全部建成人口15万以上、建成区规模15KM2以上的中小城市，形成以宿城为中心、以四个县城为次中心的城市发展格局。

——推动区域协调发展，优化生产力空间布局

坚持突出重点、分类指导的原则，加快县域经济发展，鼓励和支持各县区发挥优势，突出特色，加快构建“一核两轴”区域发展格局。“一核”即以宿城为核心，打造宿城经济圈。充分发挥宿城政治、经济、文化、交通等优势，加大招商引资力度，大力发展加工制造业、煤电产业和城市服务业，促进生产要素快速集聚，形成全市经济发展制高点。“两轴”即打造萧砀和灵泗经济板块。萧砀经济板块，主要发挥濒临徐州经济圈的地缘条件和煤炭、水果等资源优势，大力发展煤电产业、水果加工和旅游业，加快形成一批产业聚集区，发挥好示范带头作用。灵泗经济板块，主要依托丰富的农业和畜牧资源，积极发展农副产品精深加工和劳动密集型加工配套产业，进一步蓄积发展能量。通过“一核两轴”的协调互动，优化资源配置和产业布局，促进县域经济振兴。

——统筹经济社会发展，构建和谐宿州

坚持发展为了人民、发展依靠人民、发展成果由人民共享，立足现有条件和财力可能，逐步加大对各项社会事业投入，着力解决关系人民群众切身利益的问题。巩固“两基”成果，大力发展职业教育、高等教育和民办教育，扩大优质教育资源，促进各类教育协调发展。完善医疗救治体系、疾病预防控制体系和卫生监督体系，建设一批重点医院和重点学科，提高医疗服务水平。加强对文化、体育设施的投入，培育壮大文化、体育产业。健全就业服务体系、社会保障体系和城乡困难群众社会救助体系，发展老龄、妇女、儿童、残疾人和慈善福利事业。加快发展循环经济，扎实推进“生态宿州”建设，构建资源节约型和环境友好型社会。建立健全各类突发事件应急机制，推进社会治安防控体系建设，提高保障生产安全、公共安全和处置突发事件的能力。深入推进民主法制建设和精神文明建设，提高依法治市水平和全社会的文明程度。

三、扎实做好2006年各项工作

2006年是实施“十一五”规划的第一年，开好头、起好步至关重要。我们必须坚持以科学发展观统领经济社会发展全局，坚持既定的发展战略不动摇，努力推进经济社会快速平稳发展，实现全市生产总值增长9.5%以上，力争实现两位数增长；财政收入增长10%；全社会固定资产投资增长25%；实际利用内资和外资分别增长20%和18%；城镇居民人均可支配收入、农民人均纯收入分别增长8%和7%；城镇登记失业率控制在4.5%以内；人口出生率控制在13‰以内。重点抓好以下七个方面的工作。

（一）强力推进对外开放

加快融入徐州经济圈。以观念融入为先导、产业接轨为核心、企业合作为主体，全面落实双方合作框架协议。加大产业协作力度，加强与徐州大企业的联系，促进产业互补发展。拓展经贸流通合作领域，积极联办名优农产品、工业品展销和项目推介会。抓好皇藏峪、大五柳等重点景区的引资开发和对外宣传策划，促进汉文化旅游、红色旅游与徐州旅游联动发展。推动基础设施对接，做好睢明高速公路前期工作，加快相连道路的改造升级，为顺畅融入创造条件。建立各县区“跟跑”考核激励机制，推动县域经济更快发展。

大力提升招商引资水平。坚持把招商引资作为经济工作的生命线，强化目标考核，推动招商引资工作日常化、规范化。进一步聚焦招商重点区域，巩固温台、上海招商成果，注重项目线索跟踪，抓好协议项目落实，同时积极开拓苏南和其他地域。进一步突出优势招商，充分挖掘现有资源、产业优势，高度重视引进煤电能源、加工制造和农副产品加工等关联度大的产业链项目。进一步调动各类企业对外合资合作的积极性，鼓励外商投资企业增资扩股。加强金融生态环境建设，改善金融服务，加大金融对经济发展的支持力度。精心举办第二届中国宿州灵璧石文化节、第三届中国宿州黄淮海地区农资交易会，搭建集聚人气商机、促进对外开放的舞台。

集中力量建设各类开发区。坚持把开发区作为全市经济发展的龙头，吸引社会资本和金融资本参与基础设施建设。继续加大宿州经济开发区投入，全面完成铁路以西路网及其配套建设，加快新划入区域的规划和整合。加大招商引资和项目建设力度，采取“一个项目、一套班子、一抓到底”的工作机制，确保新开工项目30个，建成投产20个，到位资金8亿元以上。支持县级开发区跨越式发展，力争建成省级开发区，加快埇桥、砀山开发区建设步伐，构筑经济增长新板块。

（二）全面启动工业三年倍增计划

快速扩张工业总量。紧紧围绕规模以上工业增加值三年翻一番目标，坚持以企业为主体，以项目推进为支撑，以产业升级为手段，以机制创新为动力，大力提升市直工业，做大做强重点企业，增强带动能力。加快发展县区工业，围绕特色经济，狠抓一批农业产业化、劳动密集型、加工配套型项目，增强县域工业实力。积极培育优势产业，加快形成“产业连片、规模经营”的工业聚集区。切实加强工业经济运行的综合协调，抓好生产要素的供需衔接，建立工业发展担保基金，构建中小企业、农业产业化龙头企业、招商引资企业等银企合作交流平台，着力解决企业融资难等问题，改善工业经济发展环境。在全市开展工业三年倍增活动，并出台相关激励、考核办法。

扶优扶强规模企业。按照内生一批、引进一批、嫁接重组一批的思路，确保每年都有一批企业进入规模行列。强化骨干企业支撑作用，进一步支持皖北煤电、中煤三建等驻宿大企业发挥优势，推进跨区域、跨行业、跨所有制的资源整合。鼓励骨干企业通过资产重组、技术创新等方式，实施低成本扩张。实施中小企业促进工程，培育壮大一批成长型企业。精心打造企业品牌和产品品牌，提升企业竞争力。高度重视企业家和高素质产业工人队伍建设，为工业三年倍增提供人才支持。

（三）推动农村经济社会协调发展

扎实做好新农村建设起步工作。结合宿州农业大市和农村经济发展实际，抓紧制定新农村建设发展规划。按照“实事求是、因地制宜、分类指导”的原则，搞好小城镇和村庄规划，每个县区各抓1个示范镇和10个示范村进行试点，启动“千村百镇”示范工程，确保新农村建设实现良好开局。抢抓国家建设社会主义新农村的政策机遇，积极争取支农项目和资金，加大对农业和农村的投入力度，加快实施奎濉河治理，争取启动湖洼地治理项目，抓好县乡道路通达工程、中低产田改造、农村沼气、人畜饮水安全工程建设，努力改善农村生产生活条件。以农村综合改革为重点，全面推进行政管理体制、流通体制、征地制度和农村信用社改革，落实好良种补贴、农机购置补贴、粮食直补等惠农政策，为新农村建设注入强劲动力。加强农村基层政权建设，切实提高乡村两级服务发展、管理社会、维护稳定的能力。

加快农业产业化步伐。坚持以产业化提升农业，以工业化致富农民，围绕“121”强龙工程，集中整合项目资金，重点扶持省市级农业产业化龙头企业，不断提高与农户的利益关联度。充分发挥农业资源优势，吸引省外、境外投资者来我市建立农业基地和加工企业，开发生态农业。壮大农村致富带头人队伍，大力发展一批中小型加工项目，拓展农业发展空间。加强农民技能培训和服务引导，确保全年劳务输出达到110万人。

大力发展现代农业。深入推进农业结构调整，抓好大宗农产品、畜禽产品和名特优新农副产品区域化布局，实施定位定向和规模化生产。加强农产品质量安全体系建设，健全农业标准体系、检验检测体系、质量认证体系，加大无公害食品、绿色食品品牌建设力度，培育和创建一批名牌农产品，增强农产品开拓市场的能力。充分发挥农技推广队伍的作用，加快利用先进适用技术改造传统农业，利用现代物质条件装备农业，提高农业现代化水平。

（四）着力提升“6＋３”工程

加大项目推进力度。坚持把项目进度作为衡量工作成效的重要标准，紧紧抓住关键环节，加强重大项目调度，加快工作节奏，努力形成争取一批、开工一批、竣工一批的重点项目工作机制。继续抓好华电宿州电厂一期、信诚化工扩建、希望集团万千饲料、沱河及奎濉河综合治理等在建项目，确保完成年度投资计划；千方百计落实项目建设条件，推进丰原生物化工、博洋纺织、龙王庙煤矿、泗永、睢明高速公路等一批重点项目如期开工；加强工作衔接，确保宿淮铁路、华电宿州电厂二期、林浆纸一体化、皖北粮食物流中心等一批重点项目前期工作取得实质性进展。

扎实做好项目基础工作。建立和完善项目储备机制，积极抢抓中部崛起、新农村建设等重大机遇，准确把握国家宏观政策走向和投资重点领域，在农业基础设施、基础产业、生态保护等领域，规划一批重大项目，并争取有更多项目列入省“861”行动计划。严格落实重点项目责任制，建立重大项目绿色通道和全程服务制度，逐级明确领导责任，层层抓好督查落实，及时解决项目运作过程中存在的突出问题，努力提高项目开工率和建成投产率。强化对各级干部项目知识的培训，建设一支熟悉项目、会抓项目、能抓大项目的干部队伍。

（五）加快建设中心城市

突出抓好城市基础设施建设。坚持以完善路网、提升功能为重点，完成南坪路、金海大道北段、银河三路、胜利西路、南外环连接线、高开路、淮海南路、西昌中路、宿怀南路等续建任务。开工建设西昌南路、张仙庙路北段、纺织中路、三八路北段和西昌北路延伸段，改造浍水路、汴河东路，完善铁路以西宿城主干路网，启动道东主干道路建设。落实城市绿地系统规划，实施城市绿化美化工程，分步建设沱河景观带，加大老城区拆墙透绿和见缝插绿，完成新建主次干道绿化配套。抓好高速公路出口、火车站出口和雪枫公园、市府广场等关键节点建设改造。加快小街小巷治理，改善城市居民生活环境。

着力提高城市经营和管理能力。优化配置城市非经营性国有资产，加大城市综合开发力度，逐步推进老城区机关单位和其他机构的整体搬迁，启动市区客运车站搬迁工作，积极促进房地产业健康发展，实现老城整治与新区建设双向互动。充分发挥市政设施市场功能，鼓励社会资本进入公共事业和基础设施领域，加快推进城市公交、污水处理等公用事业的市场化。完成宿州垃圾处理厂一期工程，新建一批垃圾中转站，加快城区污水管网铺设，提高垃圾、污水处理率。加强城市规划工作，确保城市建设管理健康有序。建立健全两级政府、三级管理、四级网络的管理体系，完成城市综合执法体制改革，相对集中行政处罚权，形成长效管理机制。进一步加大脏乱差治理力度，改善城市环境，提升城市文明创建水平。

（六）大力掀起全民创业热潮

放手发展非公有制经济。人民群众是创业的主体，蕴藏着巨大的创业潜力，要把人民群众加快发展的愿望转化成创业致富的巨大动力。继续深化国有集体企业改革，推进已改制企业二次改革，积极引进战略投资者和社会资本，盘活存量，扩大增量。积极推进全民创业，降低准入门槛，支持民间资本进入所有已向外资开放的行业和领域。充分发掘创业资源，支持机关事业单位人员领办各类企业，鼓励宿州籍在外人士返乡创业，不断壮大创业者队伍。依托水果、木材等资源优势，打造特色板块，着力培育民营企业集群。制定出台鼓励全民创业的相关政策，进一步落实民营经济发展的各项措施，通过舆论引导、服务推动，形成政府鼓励创业、群众自主创业、社会支持创业的浓厚创业氛围。

培育壮大文化产业和现代服务业。充分发掘民间文化资源潜力，依托灵璧石、钟馗画和萧县书画等文化资源，大力发展民营文化企业，培育专业文化市场，做大做强文化产业。发挥产业和区位优势，积极引进国际知名的商业企业，提升宿城商业档次，扩大商圈辐射力。加快实施“万村千乡”市场工程，引导流通网点向农村延伸，繁荣农村市场。注重培育金融产品、信息咨询、社区服务等新兴产业，开辟新的经济增长点。

（七）切实推进社会和谐发展

统筹发展各项社会事业。加强人才资源能力建设，建立健全人才选拔激励机制，培养一批富有创新创业能力的人才。充分发挥科技创业服务中心功能，加强技术咨询、技术转让等中介服务，扶持企业自主创新，培育科技型企业，组织实施“粮食丰产科技工程”，促进科技与经济紧密结合。组织实施好中小学危房改造，加强薄弱学校建设，全面提升教育发展水平。整顿医疗卫生秩序，提高医疗服务质量，加强公共卫生和农村卫生工作，启动新型农村合作医疗试点。办好第三届宿州艺术节，大力发展文化事业，丰富群众文化生活。深入开展全民健身运动，积极备战省十一届运动会。努力稳定低生育水平，有效解决出生人口性别比失调问题。严格落实项目建设环境影响评价，加强环境保护。认真组织第二次农业经济普查。努力做好外事侨务、机关事务、对台、气象、地震、档案、地方志等工作。

努力解决关系民生的突出问题。落实就业优惠政策，加快再就业园区建设，积极开展“爱心帮困就业”活动，确保全年新增就业岗位2万个。加大养老保险向非公有制企业、个体工商户和灵活就业人员扩面力度，进一步完善医疗、工伤、生育保险政策，实现动态管理下低保应保尽保。加强城乡社会救助体系建设，努力解决下岗职工、被征地农民、拆迁居民、受灾群众生活困难，提高农村五保集中供养率和补助标准。切实加强食品药品质量、医疗卫生质量、中小学校园管理和收费等重点领域的监管，严格落实安全生产责任制，严防重大安全事故和突发性公共事件的发生。

加强精神文明和民主法制建设。以创建全省文明城市为目标，深入开展文明行业、文明社区和文明村镇创建活动，加强社会公德、职业道德、家庭美德教育和未成年人思想道德建设。认真听取民主党派、工商联、无党派人士及社会各界的意见、建议，支持工会、共青团、妇联等群众团体依照法律和各自章程开展工作。全面启动“五五”普法。加强和改进信访工作，依法化解人民内部矛盾。大力推进“平安宿州”建设，落实社会治安综合治理各项措施，严厉打击各种刑事犯罪。认真落实党的民族宗教政策。进一步做好国防动员、民兵预备役、人防和优抚安置工作，争创全国“双拥模范城”五连冠。

四、切实加强政府自身建设

各位代表，当前宿州正处于大发展、快发展的关键阶段。抢抓战略机遇期，实现发展新跨越，既是历史赋予我们的光荣使命，也是各级领导干部的政治责任。我们要倍加珍惜人民赋予的权力，倍加珍惜难得的创业舞台，更加自觉地担当起肩负的重任。

强化服务意识，持续优化环境。各级政府及其公务人员要充分认识优化发展环境的长期性与艰巨性，坚持解放思想先行，坚决冲破束缚发展的陈规陋习和体制弊端，支持各县区对毗邻地区实施效果显著的政策实行“拿来主义”，支持各县区结合本地实际创造性落实上级政策。切实转变政府职能，巩固行政许可清理成果，加强行政效能建设，加快政府提速。深化政风、行风评议，增强针对性和实效性，真正把对机关及其工作人员形象的评判权交给群众，让基层和群众办事顺心，让投资者感到方便快捷。继续开展专项整治，严厉打击损害发展环境的行为，构建全社会信用体系，提升环境竞争力。

强化规则意识，严格依法行政。各级政府及其公务人员要把解放思想的锐气、加快发展的热情与科学严谨的态度、脚踏实地的作风统一起来，严格按照法定权限和程序行使权力，履行职责，依法实施公共服务和社会管理。重点抓好行政执法部门、公共服务领域的规范化建设，切实解决有法不依、执法不公、执法扰民、损民利己等群众反映强烈的问题。要完善重大问题决策程序，建立健全重大事项社会公示、社会听证、专家咨询和合法性审查制度，推进政府决策的科学化、民主化。各级政府都要自觉接受人大及其常委会的法律和工作监督，接受政协的民主监督，接受社会公众和舆论监督，提高政府的透明度和公信力。

强化责任意识，切实转变作风。各级政府及其公务人员要把增强责任心的过程，作为增强同人民群众感情的过程，时刻保持如履薄冰的心态，更多关注群众利益，把群众关心的小事当作大事办，把群众关注的热点作为重点抓。大力开展纪律和作风整顿，弘扬求真务实的精神和真抓实干的作风，坚持反对形式主义、官僚主义，坚决摒弃光说不做、弄虚作假，坚决克服无所用心、敷衍塞责的现象。实行行政问责制，对因行政不作为、乱作为，给群众利益造成重大损失和严重社会影响的，要严格追究相应责任，确保有权必有责，用权受监督，违法要追究，侵权要赔偿。

强化自律意识，坚持执政为民。认真贯彻落实《公务员法》，努力建设一支政治坚定、业务精通、作风过硬、人民满意的公务员队伍。各级领导干部要做到常修为政之德、常思贪欲之害、常怀律己之心，切实加强党性修养，始终保持高尚情操，真正把心思凝聚到干事创业上，把功夫下到抓落实上，把本领用在促发展上，以实际行动赢得群众的信任和支持。深入开展党风廉政建设和反腐败斗争，把解决损害群众利益的突出问题，作为党风政风建设的工作重点，严肃查处各类违法违纪案件，坚决纠正部门和行业不正之风，积极推进政府廉政建设，从源头上预防和惩治腐败。

各位代表，实现宿州跨越式发展，奋力推进宿州崛起，是时代赋予我们的光荣职责，需要全市上下同心同德、奋力拼搏、艰忍图成。让我们在中共宿州市委的领导下，励精图治，开拓奋进，全力以赴完成今年各项目标任务，为再绘“十一五”发展蓝图，建设富裕、文明、和谐的新宿州而努力奋斗！
